# 胸外科介入治疗应用前景展望

**DOI:** 10.3779/j.issn.1009-3419.2020.101.09

**Published:** 2020-06-20

**Authors:** 坚 胡, 军 陈, 越群 牛

**Affiliations:** 1 310003 杭州，浙江大学医学院附属第一医院胸外科 Department of Thoracic Surgery, the First Affiliated Hospital School of Medicine, Zhejiang University, Hangzhou 310003, China; 2 300052 天津，天津医科大学总医院肺部肿瘤外科 Department of Lung Cancer Surgery, Tianjin Lung Cancer Institute, Tianjin Medical University General Hospital, Tianjin 300052, China

以肺癌为代表的各类肺部疾病给人民生命健康和国家社会经济带来了巨大威胁。随着近年来科技和临床实践的不断进步，肺部疾病传统诊治模式下手段单一、手术创伤大、患者术后恢复缓慢等弊端愈发不容忽视^[1]^。因此，建立肺部疾病的微创诊治新体系尤为重要，而介入技术凭借其安全、精准、高效等优势成为胸外科微创诊治未来发展的一个重要方向。然而，作为一种新兴的技术手段，现阶段各类介入操作在胸外科领域的应用还存在诸多不足，许多介入手段的切实疗效仍有待进一步探索，且介入技术在肺部疾病的治疗中仍多处于辅助地位或用于晚期疾病的姑息治疗。另外，我国目前自主开展各类介入技术的胸外科中心仍占少数，介入技术在胸外科目前的诊治体系中仍未形成主流。加强对现阶段介入技术应用中不足之处的关注，大力发展介入微创杂交技术，在胸外科领域具有重要意义。

胸外科医生应用介入技术有得天独厚的自身优势，介入技术在胸外科领域的发展也有着令人期待的广阔前景^[2]^。目前胸外科已成熟开展电视辅助胸腔镜手术（video-assisted thoracic surgery, VATS）和达芬奇机器人手术，支气管镜机器人也在积极研发中，人工智能机器人、专科化手术机器人也即将问世，胸外科医生在手持“微创手术刀”的时代大有可为。以此为基础，借助介入手段，探索将内镜手术与人体自然腔道操作进行杂交的“微微创”手术术式是胸外科发展介入技术的专科优势。介入技术可以杂交内窥镜与胸腔镜进一步显露诸如淋巴结等潜在的手术盲区，通过快速现场评估技术（rapid on-site evaluation, ROSE）快速诊断平台，术中的细胞、病理诊断将得以实现，从而最终实现一站式、一体化的自然腔道无痕手术。

此外，通过由高新技术实现的淋巴结示踪技术，可以提示各相关淋巴结的侵犯、转移情况及其疑似度，为微创杂交术中淋巴结清扫时的清扫目标、清扫价值提供更加精准化、规范化的评估。值得思考的是，在肿瘤的免疫治疗时代，淋巴结作为重要的局部免疫器官，其在免疫治疗中所扮演的角色仍有待进一步探索。因此，系统性淋巴结清扫作为肺癌根治术的标准模式或将面临挑战。在此条件下，基于淋巴结示踪技术的淋巴结采样将成为肺癌精准化诊治体系中的重要依据。

目前，介入技术在肺部疾病诊治中的应用仍处于探索阶段，还存在许多不足之处；但在微创化诊治的大背景下，介入技术的重要价值应该得到重视。其将与微创技术深入融合，辅之以免疫治疗为代表的全身治疗和全程化管理策略，形成肺癌的一体化全程诊疗新体系。

高新技术新时代赋予了临床医生新手段、新目标、新使命，胸外科学科技术发展理念更新、高新技术杂交融合将促进今后早期肺癌诊疗模式的重要变革。随着探索的不断深入，介入技术将成为胸外科领域的重要组成部分，其发展未来可期。
